# *Cat-Map*: putting cataract on the map

**Published:** 2010-10-08

**Authors:** Alan Shiels, Thomas M. Bennett, J. Fielding Hejtmancik

**Affiliations:** 1 Department of Ophthalmology and Visual Sciences, Washington University School of Medicine, St. Louis, MO; 2 Ophthalmic Genetics and Visual Function Branch, National Eye Institute, National Institutes of Health, Bethesda MD

## Abstract

Lens opacities, or cataract(s), may be inherited as a classic Mendelian disorder usually with early-onset or, more commonly, acquired with age as a multi-factorial or complex trait. Many genetic forms of cataract have been described in mice and other animal models. Considerable progress has been made in mapping and identifying the genes and mutations responsible for inherited forms of cataract, and genetic determinants of age-related cataract are beginning to be discovered. To provide a convenient and accurate summary of current information focused on the increasing genetic complexity of Mendelian and age-related cataract we have created an online chromosome map and reference database for cataract in humans and mice (*Cat-Map*).

## 1. Introduction

Cataract is a light-scattering disorder of the crystalline lens that despite surgical treatment remains an important cause of visual impairment in the United States and worldwide [1,2]. Typically, cataract is acquired with age (>50 years) as a multi-factorial disorder involving complex interactions between genetic and environmental risk factors [3]. In some cases however, cataract may be inherited as a classic Mendelian disorder (~1/10,000 births), either in association with other ocular and/or systemic abnormalities or, as an isolated lens phenotype [4,5]. By contrast with age-related cataract, Mendelian forms of cataract can occur at any age, however, most have an early-onset either presenting at birth (congenital), during infancy, childhood or adolescence. Beyond age-at-onset both inherited and age-related forms of cataract are clinically heterogeneous with respect to location, size, shape, density and even color of opacity within the lens [6-8].

In 1968 an inherited form of isolated cataract, which had previously been linked to the Duffy blood group locus, became the first monogenic disorder in humans to be assigned to an autosome (chromosome 1) [9]. Since then, considerable progress has been made in mapping and identifying genes for inherited cataract mostly by linkage analysis in extended families. All three classical types of Mendelian inheritance have been described for familial cataract; however, the existence of Y-linked cataract remains to be confirmed [10]. Similarly, genetic forms of cataract are also found in laboratory mice, and many spontaneous, chemical or radiation induced, and genetically engineered (transgenic and gene-targeted) mutant strains have been described [11]. Many of the causative genes in mice are orthologs of those identified in humans, and these mutant strains provide valuable model systems to investigate human cataract.

In contrast to Mendelian cataract our understanding of the genetic determinants of complex age-related cataract is less well advanced. Candidate gene association studies are providing increasing evidence that variations in some of the genes linked with inherited forms of early-onset cataract are associated with the much more common forms of age-related cataract. However, the advent of genome-wide association studies predicts that the genetic diversity of age-related cataract will likely extend beyond known genes for inherited cataract.

In an effort to aid access to genetic information focused on cataract we have created an online chromosome map and reference database for inherited and age-related forms of cataract in humans and mice (*Cat-Map*). Genes and loci linked or associated with familial and acquired forms of primary cataract, respectively, were obtained by keyword searches of PubMed, Online Mendelian Inheritance in Man (OMIM), and other databases accessible through the National Center for Biotechnology Information (NCBI) website [12]. Inheritance pattern, family or population origin, gene mutation or variation, cataract appearance, and any associated ocular or systemic phenotypes were also noted. Gene mutations and variations were numbered according to standard nomenclature recommendations starting with the A of the ATG start-codon [13]. Genes and loci for syndromes with cataract and mouse mutants with cataract were also included. Data for each chromosome were formatted in Microsoft Excel and links provided to the EntrezGene and PubMed databases. Due to the need for regular updating of content we have not included chromosome tables with the present text. The most recent updates can be accessed at the *Cat-Map* website. Here we provide a brief overview of the genes underlying Mendelian and age-related forms of cataract in humans and mice.

## 2. Genes for Mendelian cataract

Mendelian forms of cataract are highly clinically heterogeneous and comprise a broad spectrum of genetic conditions. In a clinical setting it is pragmatic to distinguish between “syndromic” and “non-syndromic” forms of cataract based on the presence or absence of associated ocular and/or systemic abnormalities, respectively. However, it should be noted that these are relative, rather than precise, clinical terms essentially describing opposite phenotypic extremes of the cataract spectrum disorders. Some clinical overlap between syndromic and non-syndromic forms of cataract is inevitable largely because different mutations in a single gene can exhibit pleiotropic effects that result in distinct and seemingly unrelated phenotypes with variable onset, severity and course.

### 2.1 Genes for non-syndromic cataract

Non-syndromic forms of familial cataract present as an isolated or primary lens disorder in the absence of other clinically prominent phenotypes. Under slit-lamp examination non-syndromic familial cataract exhibits a diverse range of lens opacities including, total, nuclear, lamellar, sutural, and anterior/posterior polar or sub-capsular, however, there is no universally accepted classification system [6,7]. Non-syndromic cataract also exhibits considerable inter- and intra-familial variation affecting age-at-onset, rate of progression, and post-surgical visual acuity. Moreover, relatively subtle ocular signs occasionally accompany primary cataract further complicating clinical classification. These pleiotropic effects may involve the anterior segment (e.g., microcornea), refractive error (e.g., myopia), and eye movement disorders (nystagmus, strabismus, amblyopia); further highlighting the importance of the lens in normal eye development and refractive vision. Non-syndromic forms of cataract may be grouped based on the known underlying genes, which currently include those coding for crystallins, membrane/cytoskeleton proteins and a transcription factor.

### 2.1.1 Crystallin genes

Around 60 different mutations segregating in some 98 families have been identified in 10 human crystallin genes, including those coding for both α-crystallins (*CRYAA*, 21q; *CRYAB* 11q), two acidic β-crystallins (*CRYBA1*, 17q; *CRYBA4*, 22q), three basic β-crystallins (*CRYBB1*, *CRYBB2*, and *CRYBB3* all on 22q), and three γ-crystallins (*CRYGC*, 2q; *CRYGD*, 2q; and *CRYGS*, 3q). Generally, mutations in crystallin genes tend to cause nuclear or lamellar lens opacities. In addition to primary cataract, mutations in crystallin genes, particularly those in *CRYAA*, may be associated with microcornea. However, a noteworthy exception is that mutations in *CRYAB* may underlie cataract and/or myopathy phenotypes [4,5]. Overall, crystallin mutations, mostly missense, account for about 50% of non-syndromic familial cataract reported so far.

Several unusually recurrent mutations in crystallin genes are noteworthy. First, a p.P24T missense substitution in *CRYGD* has been reported in eight families, and a p.P24S missense substitution has been shown to reflect the ancestral protein sequence found in non-primate mammals [14]. Second, the apparent p.Q155X nonsense mutation in *CRYBB2* in fact results from a gene conversion event with the adjacent CRYBB2 pseudogene [15]. Interestingly, a rare case of a severely affected family member homozygous for the p.Q155X conversion mutation developed microphthalmia, microcornea, leukocoria, congenital nystagmus, and a dysplastic lens [16]. Third, a splice (g.IVS+1G>A/C) or a deletion (p.G91del) mutation segregating in each of five families has been detected in *CRYBA1* (17q), which encodes two acidic-beta crystallins (CRYBA3 and CRYBA1). Finally, recurrent arginine substitutions are common in *CRYAA*, *CRYGD*, and *CRYGC* [4,5].

In cataract mouse strains, over 30 spontaneous or induced mutations have been reported in ten crystallin genes. Over half of these strains carry mutations in six crystallin genes (*Cryaa*, *Cryba1*, *Crybb2*, *Crygc*, *Crygd*, and *Crygs*) that are syntenic with human counterparts linked with cataract, thereby providing relevant mouse models for human cataract [11]. The remainder of the crystallin mutant strains, however, serves to highlight species differences between humans and mice. Whereas, all seven Cryg genes harbor mutations in mice (*Cryga-f*, *Crygs*); mutations in humans are restricted to *CRYGC*, *CRYGD*, and *CRYGS*. Moreover, two γ-crystallin genes in the mouse (*Cryge*, *Crygf*) are represented by pseudo-genes in humans (*CRYGEP1*, *CRYGFP*). Finally, mice null for *Cryaa* and *Crybb2*, but not *Cryab*, also develop cataract as do transgenic or knock-in mice harboring several humanized mutations in *Cryaa* (p.R116C, p.R49C) [17,18].

### 2.1.2 Genes for membrane or cytoskeleton proteins

Mutations in at least ten other genes that encode membrane proteins or cytoskeletal proteins have been linked with about 35% of non-syndromic cataract, mostly with autosomal dominant inheritance [4,5]. Mutations in many mouse counterparts of these human genes have also been reported [11].

### 2.1.2.1 Connexin genes

The genes for gap-junction proteins (or connexins) alpha-3 (*GJA3*, 13q) and alpha-8 (*GJA8*, 1q) harbor over 34 different mutations (mostly missense) segregating in about 38 families. Notably, one of the mutations in *GJA8* (p.P88S) underlies the historically important “zonular pulverulent” cataract in an English family that was first linked to chromosome 1 in 1968 [19]. Similarly, a mutation in *GJA3* (p.L11S) underlies the unusual “ant-egg” cataract described in a Danish family [20]. Mutations in connexin genes are often associated with pulverulent (dust-like) nuclear opacities and, occasionally, *GJA8* mutations are associated with microcornea. Combined, *GJA3* and *GJA8* mutations account for about 20% of non-syndromic familial cataract. In mice, homozygous loss of *Gja3* or *Gja8* results in cataract, whereas, heterozygous loss does not [21,22].

### 2.1.2.2 Genes for major intrinsic proteins

Mutations in the genes for major intrinsic protein (*MIP*, 12q), a member of the aquaporin family of water channels, and lens intrinsic membrane protein-2 (*LIM2*, 19q), a member of the PMP22/claudin family of cell-junction proteins, account for about 5% of familial cataract. All seven mutations in *MIP* are associated with autosomal dominant cataract, whereas, both mutations in *LIM2* underlie autosomal recessive cataract. In mice, heterozygous loss of *Mip* is sufficient to trigger cataract, whereas, homozygous loss of *Lim2* is required for cataract development [23,24].

### 2.1.2.3 Genes for other membrane-associated proteins

Several unexpected genes involved in membrane-associated signaling or transport processes are causative for about 5% of non-syndromic familial cataract. These include genes encoding: transmembrane protein-114 (*TMEM114*, 16p) [25], a sequence homolog of *LIM2* with unknown function; chromatin modifying protein-4B or charged multi-vesicular protein-4B (*CHMP4B*, 20q) [26], a core component of the endosome sorting complex required for transport-III (ESCRT-III); and EPH receptor A2 (*EPHA2*, 1p) [27-30], a key component of the Eph-ephrin bidirectional signaling pathway. In mice, no mutations in *Tmem114* have been reported. Mice null for *Epha2* develop cataract, whereas, *Chmp4b* null mice are embryonic lethal [29,31].

### 2.1.2.4 Genes for cytoskeleton proteins

Mutations in the genes for beaded-filament structural protein-1 (*BFSP1*, 20p) and protein-2 (*BFSP2*, 3q), which encode intermediate filament-like cytoskeletal proteins, constitute about 4% of familial cataract. A recurrent in-frame deletion mutation has been found in *BFSP2* (p.E233del). Recently, missense mutations in the vimentin gene (*VIM*, 10p) have been associated with cataract in humans and mice [32,33]. Surprisingly, homozygous loss of *Vim*, *Bfsp1*, or *Bfsp2* is not sufficient to trigger overt cataract in mice, and several commonly used wild-type strains (e.g., 129, FVB) have been found to carry a naturally occurring deletion mutation in *Bfsp2* that is associated with a subtle, progressive loss of optical quality [34-38].

### 2.1.3 Transcription factor genes

Mutations in the gene for heat-shock transcription factor-4 (*HSF4*, 16q) underlie about 6% of non-syndromic familial cataract. A missense mutation in *HSF4* (p.R119C) segregates in the historically important Marner cataract family [39]. Mice null or mutant for *Hsf4* also develop cataract [11]. Whereas, mutations in *HSF4* appear to be restricted to an autosomal dominant or recessive cataract phenotype, mutations in several other transcription factors are usually associated with cataract plus other significant ocular defects (see below).

### 2.2 Genes for syndromic cataract

Syndromic forms of cataract present as a secondary or variably associated symptom of a genetic syndrome or metabolic disorder that features other defining ocular and/or systemic abnormalities. Under slit-lamp examination syndromic cataract shows variable phenotypes similar to those of non-syndromic opacities (e.g., sutural, posterior sub-capsular) that in some disorders constitute part of the differential diagnosis (e.g., Werner syndrome on 8q, Nance-Horan syndrome on Xp, and Lowe syndrome on Xq). The underlying genetic mechanisms are diverse and include: chromosome abnormalities (e.g., Down syndrome on 21), triplet repeat-disorders (e.g., myotonic dystrophy on 19q), loss-of-heterozygosity (e.g., neurofibromatosis type-2 on 22q), mitochondrial disorders (e.g., myopathy, encephalopathy, lactic acidosis and stroke-like, or MELAS, syndrome), and genetically complex disorders such as diabetes mellitus [40].

Other ocular defects associated with syndromic cataract vary widely affecting the optic nerve, retina, vitreous body, and anterior segment, including; Norrie disease (Xp), gyrate atrophy (10q), optic atrophy-3 (19q), aniridia/Peters anomaly (11p), and Stickler syndrome type-1 (12q). In particular, mutations in several genes for transcription factors including the homeobox genes, *PAX6* (11q), *FOXE3* (1q), *PITX3* (10q) and *VSX2* (14q), and the bZIP transcription factor V-MAF avian musculo-aponeurotic fibrosarcoma oncogene homolog (*MAF*, 16q) underlie cataract plus anterior segment developmental disorders and microphthalmia sometimes associated with secondary glaucoma [41]. In one family with autosomal dominant posterior polar cataract, two siblings homozygous for a deletion mutation in *PITX3* exhibited severe microphthalmia and neuro-developmental abnormalities [42]. Mice with mutations in these transcription factor genes also inherit significant ocular phenotypes including; small-eye (*Pax6/Sey*), dysgenetic lens (*Foxe3/dyl*), aphakia (*Pitx3/ak*), eyeless (*Pitx3/eyl*), and ocular retardation (*Vsx2/or-J*) [11,41]. In addition, mice homozygous for the *eyl* allele of *Pitx3* exhibit symptoms like Parkinson’s disease in humans [43].

Cataract has been associated with several relatively mild systemic defects that manifest after standard blood or urine laboratory-tests, and serve to highlight the sensitivity of the lens to certain metabolic stresses. Autosomal recessive galactokinase-deficiency cataract results from mutations in the gene coding for galactokinase-1 (*GALK1*, 17q), the first enzyme in galactose metabolism [44]. *GALK1* mutations affect the enzyme coding region resulting in decreased red blood cell galactokinase activity. Autosomal dominant hyperferritinemia-cataract syndrome results from non-coding mutations in the gene for ferritin light chain (*FTL*, 19q), an iron-storage protein [45]. Specifically, these non-coding *FTL* mutations are, confined to the iron response element (IRE) located upstream of the coding region, and they result in increased serum ferritin levels. Mutations in the gene for glucosaminyl (N-acetyl) transferase-2 (*GCNT2*, 6p), a blood-group glycosylation enzyme, underlie autosomal recessive cataract associated with the adult i blood-group phenotype particularly in Japanese and Taiwanese populations [46,47]. Finally, mutations in the gene for solute carrier family-16A member-12 (*SLC16A12*, 10q), a mono-carboxylic acid transporter, underlie autosomal dominant juvenile cataract plus microcornea and renal glucosuria [48]. In mice, no mutations in the IRE sequence of *Ftl* have been reported. Mice null for *Gcnt2* do not develop early-onset cataract, and mice null for *Galk1* do not develop cataract unless they are also transgenic for human aldose reductase [49,50].

Cataract can also present as one aspect of a constellation of relatively severe systemic abnormalities including neurologic disorders (e.g., Cockayne syndrome on 5q and 10q, Walker-Warburg syndrome on 9q and 14q, and Smith-Lemli-Opitz syndrome on 11q). Autosomal dominant forms of systemic cataract include: neurofibromatosis type-2 (*NF2*, 22q), myotonic dystrophy (*DMPK/SIX5*, 19q), Marfan syndrome (*FBN1*, 15q), and brachio-oto-renal-syndrome-1 (*EYA1*, 8q). Notable autosomal recessive forms of systemic cataract include; inborn errors affecting galactose metabolism (*GALT*, 9p; *GALE*,1p) and cholesterol biosynthesis (*DHCR7*, 11q; *CYP27A1*, 2q), the premature aging (progeroid) disorders, Werner syndrome (*WRN*, 8p) and Rothmund Thompson syndrome (*RECQL4*, 8q), and the spectrum of peroxisomal biogenesis disorders, which include Zellweger syndrome, neonatal adreno-leuko-dystrophy and rhizomelic chondroplasia punctata type-1 (1p, 1q, 6q, 7q, 12p, and 22q).

Finally, the X chromosome alone harbors over 15 syndromic forms of cataract including; the renal disorders - Lowe’s syndrome (*OCRL*) and Alport syndrome (*COL4A5*), the lysosomal storage disorder - Fabry’s disease (*GLA*), and the Nance-Horan cataract-dental syndrome (*NHS*). Recently, mutations underlying full-blown NHS have been predicted to result in the absence of functional NHS protein (effectively a null), whereas, copy number variations (CNVs) in the NHS gene result in the less severe allelic phenotype of isolated X-linked cataract (CXN) [51]. Mice mutant for *Nhs* also develop cataract [52].

### 2.3 Novel genes for Mendelian cataract

*Cat-Map* includes at least 16 “orphan” loci for inherited forms of cataract at which the underlying genes remain to be discovered. These include about eleven loci for autosomal dominant cataract (1p, 1q, 2p, 2q, 3q, 14q, 15q, 17p, 17q, 19q, and 20p) [53-65], four loci for autosomal recessive cataract (3p, 7q, 9q, and 19q) [66-70], and one for X-linked cataract (Xq) [71]. In addition, at least 13 genes harboring spontaneous or targeted mutations have been associated with a lens or cataract phenotype in mice but not yet in humans (*Ankb*, *Bin3*, *Dock5*, *Dnase2b*, *Efna5*, *Gjf1*, *Gpr161*, *Gpx1*, *Hip1*, *Nrcam*, *Prox1*, *Six5*, and *Sparc*) [72-84]. Interestingly, none of these mouse genes are syntenic with the orphan cataract loci in humans. Genotype-phenotype discrepancies between mice and humans are likely to be found. For instance, mice lacking *Six5* develop cataract [82,83], whereas, mutations in human *SIX5* are associated with branchio-oto-renal syndrome type-2 [85]. Nevertheless, the combined list of orphan loci and mouse mutants suggests that a substantial number of genes for Mendelian forms of cataract remain to be discovered.

## 3. Genes for age-related cataract

Age-related cataract usually presents after the 4th decade and based on slit-lamp examination may be divided into three clinical types referred to as; nuclear cataract, cortical cataract, and posterior sub-capsular cataract. Each can occur in isolation or in combination (mixed cataract), and may progress to total opacification of the lens.

Genetic epidemiological studies of affected twins and siblings predict that genetic risk factors may account for 14%–48% of the heritability for nuclear cataract, and 24%–75% of the heritability for cortical cataract [86-91]. Overall, age-related cataract is less phenotypically variable than Mendelian forms of cataract, however, the genetic complexity of the former remains largely unknown.

Intuitively, genes underlying Mendelian forms of cataract represent plausible candidates for genetic determinants of age-related cataract [92]. So far, variations in at least eight genes linked with inherited cataract have been associated with age-related cataract. These include *EPHA2* (1p), *GJA8* (1q), *GALT* (9p), *SLC16A12* (10q), *HSF4* (16q), *GALK1* (17q), *FTL* (19q), and *CRYAA* (21q) [27,29,93-99]. Notably however, the triplet nucleotide (CTG)_n_ repeat expansions underlying adult-onset cataract associated with myotonic dystrophy do not represent a significant risk factor for age-related cataract in the general population [100].

Finally, variations in at least ten other genes not directly associated with inherited cataract have been tentatively implicated in age-related cataract. These include genes that function in antioxidant metabolism (*GSTM1*, 1p; *GSTT1*, 22q) [101,102], xenobiotic detoxification (*NAT2*, 8p) [103], DNA repair (*ERCC2*, 19q) [104], folate metabolism (*MTHFR*, 1p) [105], lactose metabolism (*LCT*, 2q) [106], RNA demethylation (*FTO*, 16q) [107], lipid/cholesterol transport (*APOE4*, 19q) [108], kinesin/microtubule motor transport (*KLC1*, 14q) [109], and one of unknown identity (*ARCC1*, 6cen) [110].

## 4. Summary and outlook

Currently, *Cat-Map* totals almost 200 genes and loci for Mendelian and age-related forms of human cataract spread across all 22 autosomes and the X-chromosome. At least 35 independent loci, including over 20 known genes, have been identified for non-syndromic cataract segregating most often as an autosomal dominant trait with high penetrance in over 190 families worldwide. As much as 70% of autosomal dominant cataract may be accounted for by missense coding mutations in the genes for crystallins, particularly *CRYAA*, *CRYBB2*, and *CRYGD*, and connexins (*GJA3*, *GJA8*). *Cat-Map* also includes over 130 genes and loci for syndromic forms of cataract, many associated with neurologic abnormalities, and over 70 mouse mutants with cataract, some of which point to novel genes for human cataract. Finally, variations in at least eight genes underlying genetic forms of cataract (*EPHA2*, *GJA8*, *GALT*, *SLC16A12*, *HSF4*, *GALK1*, *FTL*, and *CRYAA*), and at least 10 other diverse genes have been associated to varying degrees with age-related cataract. Collectively, however, the currently implicated genes are likely to account for a relatively small proportion of the genetic risk for age-related cataract.

The extensive clinical and genetic heterogeneity of Mendelian and age-related forms of cataract limits efforts to make informative genotype-phenotype correlations. Mutations in the same gene may result in different phenotypes. For example, *CRYAA* mutations can result in cataract +/− microcornea, whereas, *CRYAB* mutations can result in cataract and/or myopathy. Similarly, mutations or variations in *EPHA2*, *HSF4*, and *CRYAA* are each associated with autosomal dominant, autosomal recessive and age-related forms of cataract. By contrast, mutations in different genes can result in similar phenotypes. For example, mutations in multiple genes have been associated with cataract plus microcornea (e.g., *MAF*, *CRYAA*, and *GJA8*), posterior polar cataract (e.g., *PITX3*, *EPHA2*, *CHMP4B*, and *NF2*), or sutural cataract (e.g., *BFSP2*, *CRYAB*, and *NHS*). Further, an increasing number of genes have been associated with age-related cataract especially cortical or mixed types (e.g., *EPHA2*, *HSF4*, and *CRYAA*). It is likely that the clinical and genetic heterogeneity of cataract will continue to expand particularly in cases where a non-lens phenotype may present long after the diagnosis of early-onset cataract. Recently, variations in *CRYAB* and *PITX3* have been associated with increased susceptibility to multiple sclerosis and Parkinson’s disease, respectively [111,112], raising awareness about the possibility of acquired neurodegenerative conditions in certain patients with early-onset cataract. Such observations support the notion that a gene-based system will enable a more informative clinical classification of the cataract spectrum disorders.

In the future, family-based linkage studies and case-control association studies in different populations will continue to identify, test and validate genetic determinants of inherited and age-related forms of cataract. Moreover, the advent of next-generation sequencing techniques capable of rapidly deciphering genomic variation in large numbers of individuals will provide powerful insights regarding the molecular genetic basis of cataract; including gene-gene and gene-environment interactions. Ultimately, a comprehensive understanding of the genomic determinants of cataract, coupled with improved phenotyping of animal models, will not only enhance understanding of the molecular biology of lens development and aging but also may translate into non-surgical treatments for cataract, or even lifestyle interventions (e.g., diet) that help to prevent cataract.

In summary, *Cat-Map* provides a curated portal to access peer-reviewed literature (PubMed) and bioinformatics (EntrezGene) focused on the genetic causes of cataract in humans and mice, and a gene-centric basis to aid clinical classification of cataract.
